# A Case of Diabetic Ketoacidosis in a Patient on an SGLT2 Inhibitor and a Ketogenic Diet: A Critical Trio Not to Be Missed

**DOI:** 10.1155/2020/8832833

**Published:** 2020-08-13

**Authors:** Samantha Steinmetz-Wood, Matthew Gilbert, Katherine Menson

**Affiliations:** Department of Medicine, Larner College of Medicine at the University of Vermont, 111 Colchester Ave, Burlington, VT, USA

## Abstract

Results from major clinical trials have shown significant cardiorenal-protective benefits of SGLT2 inhibitors in patients with type 2 diabetes (T2DM), leading to increased popularity. A rare but serious side effect of SGLT2 inhibitors is euglycemic diabetic ketoacidosis (EDKA), which presents more covertly but has been described. Identification and report of modifiable risk factors would be an important step in helping clinicians appropriately counsel patients. In this case report, we present DKA in a patient on an SGLT2 inhibitor and ketogenic diet (KD). A 47-year-old male with a history of poorly controlled T2DM on metformin and empagliflozin presented to the emergency department (ED) with several days of pharyngitis, dyspnea, emesis, abdominal pain, and anorexia. Of note, one month prior to this event, he presented to the ED with malaise and was found to have an anion gap of 21, a bicarbonate level of 13 mmol/L, a pH level of 7.22, 3+ ketonuria, and a glucose level of 7 mmol/L (127 mg/dl). Additional workup was negative, and findings were attributed to his KD. His use of empagliflozin was not identified on his medication list. At second presentation, the patient was tachypneic and tachycardic and had mild abdominal tenderness. Labs revealed anion gap 28, bicarbonate 5 mmol/l, pH 6.94, 3+ ketonuria, glucose 14.9 mmol/L (269 mg/dl), and beta-hydroxybutyrate 8.9 mmol/L. The patient was diagnosed with DKA and was treated accordingly. With closure of anion gap, the patient was transitioned to insulin and metformin, and his empagliflozin was discontinued indefinitely. Before prescribing this medication class, physicians should inquire about low-carbohydrate diets given the higher risk for DKA, though knowledge of this risk is still not widespread.

## 1. Introduction

Recent results from major clinical trials such as CANVAS, CREDENCE, EMPA-REG OUTCOME, and DAPA-HF have shown significant cardiovascular and renal protective benefits of SGLT2 inhibitors [1–4]. These findings have led to increased use of this class of medication in patients with type 2 diabetes (T2DM). Although reported as rare, a serious side effect of SGLT2 inhibitors is diabetic ketoacidosis (DKA), which can present more covertly with euglycemic DKA and has been described numerous times. There are some thoughts about patients that might be at greater risk for developing this condition such as longer duration of diabetes, insulin deficiency, or even possible variants of the SGLT molecule [5, 6]. Identification of additional modifiable risk factors such as special diets would be an important step in helping clinicians choose which patients should avoid using this medication. In this case report, we present a case of profound DKA in a patient on an SGLT2 inhibitor on a ketogenic diet.

## 2. Case Presentation

A 47-year-old male, with a 10-year history of T2DM, presented to the emergency department (ED) with several days of sore throat, dyspnea, nonbloody emesis, abdominal pain, and poor oral intake. He denied any fever, chills, cough, chest pain, or diarrhea. He had a urinary tract infection (UTI) 2 weeks prior, which had resolved with a course of antibiotics. Over several months, he had made significant changes in his diet which resulted in a 60 lb weight loss. The patient reported that he was following an Atkins or ketogenic diet. In addition to diet control, he had been on metformin and empagliflozin started approximately 5 months ago. His last hemoglobin A1c was 76 mmol/dl (9.1%). Of note, one month prior to this presentation, he presented to the emergency department with weakness, intermittent chest discomfort, and shortness of breath with an anion gap of 21, a bicarbonate level of 13 mmol/L, a pH level of 7.22, and 3+ urinary ketones with a glucose level of 7 mmol/L (127 mg/dl). Cardiac workup was negative, lactate was normal, and liver enzymes and D-dimer were within normal limits. The patient was given 1 L fluid bolus with some improvement. Findings were attributed to a keto diet. The patient was discharged with recommendations to increase carbohydrate intake for 2 weeks and repeat electrolytes. His use of empagliflozin was not identified on his medication list in the emergency department nor was it mentioned in the ED notes. His repeat labs one week later did result in closure of his anion gap to 10. When this second presentation to the emergency department 24 days later, he was afebrile, tachypneic with a respiratory rate of 30, and tachycardic with a heart rate of 130 beats per minute, blood pressure was 160/89 mmHg, and SpO2 was 100% on room air. Other than respiratory discomfort, the exam only showed mild abdominal discomfort to palpation. Labs were significant for an anion gap of 28, a pH level of 6.94, with a bicarbonate level of 5 mmol/L, 3+ urinary ketones, a beta-hydroxybutyrate level of 8.9 mmol/L, and a glucose level of 14.9 mmol/L (269 mg/dl), as well as acute kidney injury with a creatinine level of 107 umol/L (1.21 mg/dl). His glutamic acid decarboxylase antibody assay ordered during this admission eventually returned negative. His C-peptide was 0.77 nmol/L (2.3 ng/ml). Cardiac workup was negative. CT scan did reveal possible aspiration pneumonitis. The patient was diagnosed with diabetic ketoacidosis, received several litres of fluid, started on insulin drip, and admitted to the medical ICU. With closure of anion gap, on the following day, the patient was transitioned to insulin basal bolus regimen, and metformin was eventually restarted. His empagliflozin was stopped indefinitely. Hospital course was also complicated by development of a new UTI, and the patient was treated with ceftriaxone/cephalexin. Finally, he was deemed medically stable for discharge with plans to follow up with endocrinology and his primary care provider.

## 3. Discussion

Our case illustrates a case of profound DKA of a patient on an SGLT2 inhibitor and following a ketogenic diet. Interestingly, our patient also developed a UTI twice in the same month, which is another reported side effect of this medication. Randomized control trials studying SGLT2 inhibitors have indicated DKA to be a rare side effect, with an estimated incidence rate of DKA varying from 0.13 to 0.76 events per 1000 patient years [6].

On review of the literature, this is not the first case of DKA in a patient on an SGLT2 inhibitor following a ketogenic or low-carbohydrate diet [7–10]. Another case reports a similar story of a 44-year-old man who had been on sitagliptin, metformin, and an Atkins diet, and 3-4 days after starting canagliflozin, he developed euglycemic DKA which was first missed by the ED and later recognized by his primary internist [10]. As in our case, our patient had already presented with euglycemic DKA to the ED, which was diagnosed as dehydration and starvation ketosis. Luckily, at that time, he recovered by one week with change in diet. Later, he presented with profound dehydration and severe anion gap metabolic acidosis with a bicarbonate level of <5 mEq/L, requiring inpatient critical care. Recognition of the association of this medication with euglycemic DKA and asking about medications is most important.

Studies have measured patients on SGLT2 inhibitors to have low-grade asymptomatic ketonemia [11, 12], and in a Japanese multicenter, randomized, 3-arm parallel comparative study, patients with T2DM assigned to low-carbohydrate diet on luseogliflozin had significantly higher serum ketone bodies than their study counterparts on higher carbohydrate diets [12]. Although ketonemia may be perceived as a negative effect of SGLT2 inhibitors, this seems to be their main mechanism of benefit. SGLT2 inhibitors are thought to act through calorie-restriction mimicry and by a proketogenic effect [13]. This also decreases the insulin to glucagon ratio, thereby improving insulin sensitivity. Ketone bodies are additionally a more efficient source of fuel for cardiac muscle [13].

A Canadian clinical literature review reviewed 46 cases of SGLT2-associated DKA and found that the most common precipitants of DKA were inappropriate insulin reduction or cessation, surgery, excessive alcohol intake, excessive physical exercise, and dietary restriction such as low-carbohydrate or reduced intake [14]. As SGLT2 inhibitors themselves mimic a low-carbohydrate diet and promote ketosis, it follows that the pairing of SGLT2 inhibitors and a ketogenic diet, in addition to stressors, may more easily precipitate a ketoacidotic state.

## 4. Conclusion

Before prescribing this medication class which is now starting to be more widely prescribed, physicians should ask whether patients are following low-carbohydrate diets as this likely puts them at higher risk for DKA. Knowledge of this risk is still not widespread.
